# Supplementary material for: Beyond active learning: Using 3-Dimensional learning to create scientifically authentic, student-centered classrooms
Source: PLoS One. 2024 May 31;19(5):e0295887. doi: 10.1371/journal.pone.0295887 (PMC11142574; doi:10.1371/journal.pone.0295887)
Supplement: S1 File — (PDF) [file pone.0295887.s001.pdf]

readme document for the organization of the ‘LOP data 3D vs Active.xlsx’ file.

Course numbers are not included, as they could potentially be used to identify specific instructors.

The file is organized column-wise, showing meta information for the class/segments. The first row is a descriptive comment line indicating what each column stores.

Rows: each row is a single class segment.

Columns

A. Class Observation ID number
B. Discipline
C. Project Year
D. Segment Start Time [minutes]
E. Segment End Time [minutes]
F. Practice Centeredness Code (Instructor/Student/N/A)
G. Scientific Practice Code
H. Core Idea Code
I. Crosscutting Concept Code

J - O.Teaching Activity Columns

Dimension Codes:

- Scientific Practices
  - 0: None
  - 1: Asking Questions
  - 2: Developing and Using Models
  - 3: Planning Investigations
  - 4: Analyzing and Interpreting Data
  - 5: Mathematical and Computational Thinking
  - 6: Constructing Explanations or Argumentation from Evidence
  - 7: Evaluating Information

- Core Ideas
  - CEM
    - 0: None
    - 1: Electrostatics and bonding interactions
    - 2: Atomic and molecular structures and properties
    - 3: Energy
    - 4: Change and stability in chemical systems
  - PHY
    - 0: None

        - 1: Interactions can cause changes in motion
        - 2: Energy
        - 3: Exchanges of energy increase total entropy
        - 4: Interactions are mediated by fields
        - 5: Energy, momentum, and information can be transferred without a net transfer of matter
    - BS
        - 0: None
        - 1: Chemical and physical basis of life
        - 2: Matter and energy
        - 3: Cellular basis of life
        - 4: Systems
        - 5: Structure and function
        - 6: Information exchange and storage
        - 7: Evolution

- Crosscutting Concepts
    - 0: None
    - 1: Patterns
    - 2: Cause and Effect
    - 3: Scale
    - 4: Proportion and Quantity
    - 5: Systems and System Models
    - 6: Energy and Matter Flows
    - 7: Structure and Function
    - 8: Stability and Change

Teaching Activity Columns
- 2 columns per activity
    a. Number of occurrences [N]
    b. Total duration of all occurrences [T, minutes]
- Activity Order:

    J. Miscellaneous
    K. Admin
    L. Lecture
    M. Interaction
    N. Clicker Question
    O. Task
