# Supplementary material for: Beyond active learning: Using 3-Dimensional learning to create scientifically authentic, student-centered classrooms
Source: PLoS One. 2024 May 31;19(5):e0295887. doi: 10.1371/journal.pone.0295887 (PMC11142574; doi:10.1371/journal.pone.0295887)
Supplement: Attachment — Submitted filename: Response to reviewers.docx [file pone.0295887.s003.docx]

# Responses to Reviewer Comments (in italics)

Reviewer #1:

Was there a supporting information file that was not included? The authors provide none of their data and the paper only contains "representative examples" of timelines. Perhaps the submission was incomplete for some technical reason?

As it stands it is impossible to review this submission based on the PLOS criteria.

*Response: We have uploaded the de-identified coding sheets, which contain the data used in this paper. However, since the original videos contain identifiable faculty we are not able to supply links to those since our IRB is predicated on individual faculty being de-identified. More timelines and data analysis are also available in our paper describing the development of the 3D-LOP, along with supplementary information files that describe the explicit coding procedure.*

## Reviewer #2:

This is an excellent manuscript and an exciting suggestion of how to move beyond active learning. My biggest suggestion is that I was left wanting a little more by way of the discussion/conclusion. I loved figure 1 but I would suggest an additional figure or table later on showing the 3DL areas and how they were specifically applied in this study. Also more discussion of ways to implement etc. I found that you all showed that this is potentially beneficial but a bit more discussion of implication s would be beneficial.

*Response: Thank you for this suggestion. We have added a new section highlighting papers that address “How to do 3DL” in different ways, including how to adapt assessments, examples of 3D curricular materials, a description of the Fellowship, and a report of how faculty were impacted. In addition, we have added a section to the discussion specifically discussing how the class that we coded as active but not 3D might have been redesigned to align with the 3DL Framework.*

## Reviewer #3:

As someone who as been following the 3D learning literature over the past couple of years, this paper is greatly appreciated and in my opnion it adds value to the existing body of research. I feel this paper should be accepted for publication, however I do have a few minor suggestions. I hope the authors find these useful and that they might help further improve the manuscript.

1) The authors report that 93 of the 417 instructional segments were identified as 3DL. I would be interested to know how many different instructors taught these 93 segments. If this can be reported, I would then be interested to see a summary of how many of these instructors were exposed to the 3DL literature and how many (if any) may have incorporated 3DL components into their course without explicitly knowing about the 3DL reform movement. In my experience discussing 3DL with colleagues in my department, many of them incorporate aspects of 3DL in their teaching without having ever been exposed to this framework prior to our discussions. If all of the 3DL segments in this study were from faculty who went through some specific 3DL training or were known to have been exposed to the 3DL literature, the authors might note in the discussion that many STEM faculty in higher ed likely value 3DL principles without necessarily being aware of 3DL reform efforts. I think this might help faculty instructors who are not immersed in the chemistry education literature (e.g., research faculty at R1 institutions), but who are doing good work in the classroom feel more valued for their efforts to push their students to engage in meaningful ("3D-esque") learning.

*Response: We agree that knowing how many of the 3D classes and segments were the result of specific exposure to 3DL would be interesting, but in practice counting the number of Fellows (and proportion of those 93 segments) is really all we can do. However, there are many ways that faculty may have been exposed to 3DL through influences outside the time and space of the Fellowship (for example, faculty who team teach in a multi -section transformed course alongside alumni of the Fellowship). More broadly, we note that the research question implicated in this comment is beyond the scope of this paper, both because of the difficulties associated with establishing (in)direct exposure to 3DL, and because our IRB commitment to anonymity.*

2) In the concluding paragraph in the Results/Implications the authors might note that incorporating 3DL in higher ed might need to be thought of in a more holistic way with respect to the four-year curriculum? For instance, though we should obviously aim to incoproate 3DL in our intro general chemistry/bio/physics courses, one could argue there are many skills that students need to learn in these courses which are then used throughout the subsquent curriculum and the prevelance of 3DL in these courses may not be as high. Perhaps there could/should be a continuum of 3DL that increases as students progress through the four-year degree program? I don't think this would change the overall argument being made in this concluding paragraph, but it might provide some valuable nuance.

*Response: We completely agree with the Reviewer that 3DL should not be confined to introductory courses, and should continue throughout the curriculum, and have attempted to clarify our beliefs. It was not our intention to imply that 3DL only applies to gateway courses, but most of the data come from such courses and they were also the initial focus of the MSU fellowship program.*

3) The authors state on pg. 19 that "...only one of them [3DL] provides a mechanism to support students’ use of knowledge in scientifically authentic ways." While I generally agree with this statement, I think some clarification from the authors might be prudent. For instance, if I employed an activity in my class that required students to apply their knowledge of time-integrated rate laws to evaluate concentration and time data to determine/evaluate the stability of two different drug molecules (SPs = Analyzing and Interpreting Data, Engaging in Argument from Evidence, and Constructing Explanations), but I didn't explicitly connect this to the 3DL core ideas or integrate an engineering pratice, would this be 3DL? If not, would this not still be using knowledge in a scientifically authentic way (I would argue yes it is)? The authors might explicitly state what they would consider to be 3DL and not 3DL, and if they were to use a more stringent defintion, they might acknowledge that active learning without 3DL could be in some sort of in-between state in which meaningful/authentic learning is still taking place but a full-fledged 3DL reform has not been carried out.

*Response: This is a good point, and in our work we have consistently stated that not every class activity is (or should be) 3D (e.g., Matz 2018), and have reiterated that here. The activity described in the Reviewer’s comment is clearly important and useful, and certainly engages students in scientific practices. The point we have tried to make is that as students develop disciplinary expertise, at some point the skills and knowledge they gain must be connected to core ideas (not necessarily in every class in every activity). The goal is to avoid a course where techniques are solely emphasized in the absence of disciplinary ideas. 3DL provides a concrete path to authentic engagement and (the current study says) is also more likely to actively engage students.*

Reviewer #4:

The manuscript explores the concept of three-dimensional learning (3DL) in STEM education, challenging traditional active learning methods by integrating three key components: scientific and engineering practices, disciplinary core ideas, and crosscutting concepts. This approach is inspired by the National Academy's 'A Framework for K-12 Science Education.' The authors have collected data from various courses to assess 3DL's impact in higher education, particularly its role in enhancing student engagement and learning effectiveness. They argue that 3DL promotes not only student engagement but also meaningful interaction with core disciplinary ideas, an aspect often overlooked in traditional active learning.

However, the manuscript could benefit from additional details in several areas. I recommend acceptance after addressing the following points:

1. Implementation of 3DL: More specific examples or case studies demonstrating 3DL's application in diverse educational settings would clarify its implementation.
2. Comparative Analysis: An in-depth comparative analysis with other educational models, especially active learning methods, would enrich the discussion, highlighting 3DL's unique advantages and challenges.
3. Quantitative Evidence: The manuscript would benefit from a broader presentation of quantitative data, such as student performance metrics or statistical analyses, to substantiate the effectiveness of 3DL.
4. Long-term Impact: Discussing the long-term effects of 3DL on students' learning outcomes and career trajectories would provide a more comprehensive view of its benefits.

*Response: While some of these suggestions are certainly fruit for further studies, we believe that they are all beyond the scope of the current paper. The paper is not intended to be a primer on 3DL - especially since we have published earlier studies on these topic.:*

*Suggestion #1. We have added a short section to point readers to our published papers that help clarify implementation. Case studies of active and 3DL classes would be an interesting study, but are beyond the scope of this paper.*

*Suggestion #2. We have added a section to the discussion specifically addressing how the class that we coded as active but not 3D might have been redesigned to align with the 3DL Framework.*

*Suggestion #3. We have published extensively on comparisons of 3DL courses and more traditional (be they “active” or “traditional lecture”) and have discussed several of them including r citations 20-31.*

*Suggestion #4. It is very difficult to track students as they move through multiple curricula and majors at such a large university. We certainly do not have the resources to follow students into their career trajectories. We do have data on students as they move through 4 semesters of chemistry, and we have added a short section indicating that students from the large enrollment 3D general chemistry courses do as well or better than students from more selective (honors and majors) courses in the subsequent organic chemistry courses.*

## Reviewer #5:

The three strands of “Three-Dimensional Learning (3DL)” are Scientific Practices, Core Ideas, and Crosscutting Concepts. 3DL is a useful framework for student learning in STEM classrooms and, as argued by the authors, is more precisely defined than “active learning.” This paper addresses two questions: (i) what does a class look like when it is engaged in 3DL, active learning, or combinations of the two? And (ii) how are 3DL and active learning related to one another?

The paper summarizes the analysis of 417 video-recorded segments of 82 class meeting sessions in the introductory courses in biology, chemistry, and physics. Diagrams of each of four different instructor pedagogical approaches are shown: active but not 3D, active and 3D, 3D but not active, and neither active nor 3D. Analysis indicates that 3D instruction is moderately associated with active learning, but not the reverse.

I have three comments:

1. While it appears that the authors believe that there is an obvious difference between active learning vs. 3DL, it is not clear how the differences manifest in the practices/behaviors in the classroom. Therefore, for the non-specialist reader, it could be very helpful to illustrate the difference(s). To illustrate the differences as they are operationalized, it would be informative to show how the same material can be presented in two different ways, rather than the present version which reports observations on four different topics and fields. For example, in the first segment (meiosis) diagramed, what could the instructor change so that the class would be an example of 3D or 3D+active?

2. The paper appears to imply that a course that uses 3D is a better way to engage the students than a course that only uses active learning. If my interpretation is correct, then it would be helpful to clearly explain (a chart or table?) the differences and their benefits and shortcomings.

*Response (to both comments 1 & 2): We thank the reviewer for this useful suggestion. A paragraph and associated table has been added to the revised discussion to address this comment (and Reviewer #2’s similar suggestions). By using the example “active but not 3D” class meeting that was presented in one of the timelines as an anchor, we illustrate the type of instructional re-framing that is required to provide students opportunities to engage with 3DL. We believe these comments have led to greater clarity in the revised manuscript in our effort to highlight that such a transformation is not a shift or tweak in an instructional strategy, but a rethinking of what is valued as important that students know and do.*

3. In several places, the Introduction appears to dismiss or diminish the significance/efficacy of active learning. If this is the intended message, then I think it can be said once, directly, and with more details (see comment 2, above).

*Response: Our intent was not to diminish the work on active learning that has gone before, but rather to point out (as has been noted by others) that it is ill defined, and simply adding an student engagement component does not ensure deeper or more authentic learning. We hope the addition of the table and following discussion makes this clearer.*

## Reviewer #6:

Please add more references linked with the qualitative interpretation in your article.

*Response: We have added more references and more discussion in the implications section as discussed earlier.*

## Reviewer #7:

The authors address a relevant topic and an interesting research. Although the paper has merit, I would advise some revisions before the paper is published. My major concern has do to with the methodology. First, the authors make an argument on how 'active learning' is ill-defined, but they do not themselves provide a definition and use the same terminology they critique to code classrooms. I would suggest a clarification of this point, which is central, in order to sustain the work on a sound framework. From my perspective, active learning is more than using 'tasks'. If the authors want to prove that 'active learning' should not be the focus in higher education, they have to be clear on what they understand active learning is and is not.

*Response: We are not the first or the only people to who have pointed out that active learning may be in the “eye of the beholder” and means different things to different people. As we state in the paper we have used the Theobald definition(s) for student engagement (reference 5), and in our earlier paper on the development of the 3D-LOP we defined the class activities that could engage students, for example tasks, or clicker questions. We also coded whether these activities were instructor led or involved student engagement. We have also added more discussion around Table 1 and the meiosis example to make the point that we do want active learning, we just want it to be purposeful activity and 3DL provides purpose.*

Also, the authors argue that higher-order thinking is also ill-defined by quoting a study with 38 teachers in Kindergarten to Grade 9 classrooms. Given the differences between these school-levels and higher education, I think that this point needs further evidence.

*Response: We have added more references specifically focussed on higher education and provided examples of other researchers who support this viewpoint.*

I would also like to have seen a more detailed description of the methodology: when were the video recordings made (the year they started) and were the 4 years in a row or interleaved? Was the inter rater reliability assessed? Why use segments and not the entire class? How were segments defined (give examples of how 'particular topic' can be identified by coders; given that this is your ‘unit’ of observation, we need to be given information on how they can be identified)? Was there a minimum time established for each segment? What was the interval of time between the bigger segment and the smallest one? Time can also be an important variable when active learning or 3D in put into place.

*Response: The methodology we used to analyze the videos is explicitly treated in our publication on the development of the 3D-LOP. As we discuss, the videos we used for this paper are not those used for the development of the protocol, but they were analyzed in the same way. We have tried to clarify this approach in the manuscript. The videos were all recorded during four years in a row.*

Finally, I do not think that the title of paper is clear. The paper does not provide evidence that active learning does not promote student-centered classrooms or that it is not scientifically authentic, as the title may us lead to believe.

*Response: We respectfully disagree with the Reviewer. We explicitly state that active learning is central to student centered classrooms, but as it stands it is ill defined. We are offering a theory and evidence-based approach to learning, and our point here is that by engaging in 3DL we also tend to support student engagement or student centered classrooms. We show evidence that student centered classrooms are not necessarily engaging students in the practices of science.*

Correct the typo in line 253.

*Response: Done*

## Reviewer #8:

Thank you for this fine presentation of 3DLearning.

Students who are able to learn STEM concepts, effectively communicate their newly acquired knowledge either in a group setting or individually and then succesfully apply what has been learned to a new situation or problem engenders excitement in the steps toward mastering a subject. Such STEM learning at the high school level or in the early college years would decrease the number of students with low grades or course withdrawal because learning becomes fun and with purpose. Passion for scientific inquiry in chemistry, biology or physics will be iginited if students understand the why and the how to apply their newly gained knowledge in the world in which they find themselves. Thus, the connection between a student's enjoyment of learning a STEM subject and 3DLearning might be of interest to those reading this manuscript. There is no substitute for a professor or teacher who loves their subject, and can effectively impart that love to his or her students.

*Response: While we agree with this reviewer, we believe that this comment is beyond the scope of this paper.*
