# Supplementary material for: Beyond active learning: Using 3-Dimensional learning to create scientifically authentic, student-centered classrooms
Source: PLoS One. 2024 May 31;19(5):e0295887. doi: 10.1371/journal.pone.0295887 (PMC11142574; doi:10.1371/journal.pone.0295887)
Supplement: Attachment — Submitted filename: Respnse to reviewers beyond active R2.docx [file pone.0295887.s004.docx]

PONE-D-23-39689R1

Beyond Active Learning: Using 3-Dimensional Learning to Create Scientifically Authentic, Student-Centered Classrooms

PLOS ONE

**Responses to Reviewers (in italics)**

**Reviewer #1:** The authors have addressed my concerns about data availability.

This is now a very useful and technically sound manuscript that offers the following significant advances:

- demonstrates the practical utility and reliability of the 3D-LOP protocol for coding videos of instruction
- shows that active learning and 3DL can be identified separately from videos
- finds that (within their dataset) active learning does not imply 3DL, but typically 3DL does imply active learning
- offers support for instructors who wish to incorporate 3DL into their teaching, and assess their 3DL using the tools described here

**Reviewer #2:** Thank you for your revisions, I believe you have addressed all of my previous comments as well as many of the comments left by other reviewers. I noticed a few very small typos etc. that you may want to correct before publication: There appears to be a missed space on line 85; a missed period on lines 173, 202, 246, and 477; and typos on lines 456 and 480.

***Response:*** *Thank you for pointing these out, we have fixed them.*

**Reviewer #3:** PONE-D-23-39689R1

Reviewer Comments:

The authors have addressed most of the concerns and suggestions from the original reviewers, though in many cases in a minimal fashion. Ultimately, I think this paper is worthy of publication in PLOS ONE in its current form, however I feel there are still a couple of significant flaws in the broader arguments the authors are making. If these are not addressed, I suspect many readers will look at this with a more critical view and the reach/impact of this paper will be hindered (especially with respect to impacting classroom practitioners, but also with educational researchers).

1. I feel the authors are conflating the 3DL learning framework with evidence-based instructional practices. It seems to me that 3DL is a framework which acts as a base/guide for building a set of course learning outcomes (particularly through the core ideas and crosscutting concepts),

***Response:*** *We agree, that 3DL is a framework that allows us to build course learning outcomes – and just as importantly their aligned assessments. Just as with POGIL or modelling approaches, this is an approach to evidence-based instruction. To remove the confusion about what we mean be evidence based instructional practices we have removed any mention of EBIPS and instead have used the term “active learning” which has emerged as a “catchall” for any kind of student engagement activity.* *Our finding here is that enacting 3DL is likely to promote student engagement in meaningful scientific practices and will likely result in active learning whereas enacting active learning without careful attention to what is expected of students will certainly engage students but there is no grantee they will be engaged in meaningful activity. This does not, of course, mean that 3DL is the only framework that can engage students in this way, and we have tried to make this point clearer in the manuscript (see below).*

and one could easily argue the scientific/engineering practices are themselves a broader set of additional content-agnostic learning objectives (e.g., developing and using models, analyzing/interpreting data, engaging in argument from evidence, etc. are things I hope my students can do by the end of my course, but they are not distinct classroom practices) Table 1 in the manuscript highlights this point, with the fact that two completely different classroom practices (e.g., “non-active” passive instructor lecture or student-centered active learning) can both lead to 3D learning.

***Response:*** *Here we must respectfully disagree with the reviewer, scientific practices such as developing and using models, analyzing/interpreting data, engaging in argument from evidence, etc. typically have particular knowledge construction goals, and as we note in the manuscript there is ample evidence in the literature that content agnostic learning objectives are unlikely to produce the outcomes desired. The 3DL framework requires that all three dimensions (Core ideas, Scientific Practices and Crosscutting concepts) be tightly integrated. The 3DL framework specifies both the content and what students should do with that content.*

I strongly encourage the authors to reconsider highlighting how different types of evidence-based practices can lead to learning outcomes that fit within the framework of 3DL. I have highlighted here three examples of classroom practices and representative publications that I would consider active, AND which aim to achieve the kind of learning outcomes described by the 3DL framework (this is by no means an exhaustive list of evidence-based practices that might promote 3DL).

PhET activities:
a) <https://pubs.acs.org/doi/10.1021/ed4005084>
b) <https://pubs.acs.org/doi/10.1021/acs.jchemed.0c00470>
c) <https://doi.org/10.1039/D1RP00086A>

POGIL:
a) <https://pubs.acs.org/doi/10.1021/bk-2008-0994.ch018>
b) <https://pubs.acs.org/doi/10.1021/acs.jchemed.0c00355>
c) <https://doi.org/10.1039/C5RP00207A>

CURES
a) <https://pubs.acs.org/doi/10.1021/acs.jchemed.1c01179>
b) <https://doi.org/10.1021/acs.jchemed.3c00570>

***Response:*** *We agree with the reviewer that explicitly using 3DL as a framework for designing instruction and assessments is not the only framework that might promote 3DL (and indeed one of the PhET activities called out here* [*https://pubs.acs.org/doi/10.1021/acs.jchemed.0c00470*](https://pubs.acs.org/doi/10.1021/acs.jchemed.0c00470)*, was designed by two of the co-authors on this paper, and guided by their work on the longstanding 3DL4US project part of which is described here).*

*We have attempted to clarify that there are other evidence-based approaches that – even though they are not explicitly designed using 3DL – do have the potential to be 3D. For example, POGIL activities typically begin with experimental data that students analyze and interpret (SEP) to make arguments (SEP) about a phenomenon (ideally linked to a core idea and a crosscutting concept). 3DL has the advantage of being more granular than a descriptor like POGIL or CURE. Its utility derives in part from this granularity. It is possible to describe a CURE or POGIL activity that engages students in a scientific practice, crosscutting concept and core idea.*

*Although PhET is not an instructional approach – the way the simulations are be used can lend them to analysis and interpretation of data, argumentation and other scientific practice.*

*Again, our intention was not to imply that there is no effective and impactful prior work, but rather to show that focusing only on student engagement, that is “active learning” without a concomitant emphasis on what we expect students to know and to do, may not lead to desired outcomes.*

2. I also continue to take issue with two statements made by the authors:

i) “Most studies on the impact of active learning on student outcomes rely either on scores on conceptual multiple choice exams or course grades, but typically little information is provided about what those grades are measuring, and whether they emphasize factual recall or use of knowledge.”

The representative PhET and POGIL articles listed above are examples of studies that use more than course grades to assess the impact of the evidence-based practices on student learning. I feel the authors are overlooking a significant amount of previous research that has been done to assess the impact of evidence-based practices on meaningful student learning, and if they choose to ignore this in the final version of this article, that will likely reduce the persuasiveness of this paper on science educators.

***Response:*** *We have attempted to make our position clearer about the studies that rely on concept tests or course grades (it is the oft cited meta studies such as Freeman et al. – PNAS, 2014, 111(23, 8410-8415). Additionally, a review of research on POGIL has this to say about the studies “We find that this body of research has typically focused on quantitative measures that broadly assess student outcomes, with fewer studies concentrating on qualitative, theory-based explanations of student learning.” (Rodrigues et al. J. Chem Educ. 2020, 97, 3506-3520)*

ii) “…but what seems clear is that while 3DL and active learning could both be considered evidence-based approaches to teaching and learning, only one of them provides a well described framework and mechanism to support students’ use of knowledge in scientifically authentic ways.”

The representative articles above were chosen because these studies also provide examples of “using knowledge in scientifically authentic ways.” If the authors were to acknowledge the various types of evidence-based practices that can/do promote 3D learning, they could further amend this statement to highlight how 3DL is a framework of learning that promotes the use of learning objectives aimed at the authentic use of knowledge, which can then be achieved either through explicit “3DL” curricula (e.g,. CLUE) or other evidence-based practices (POGIL, PhET concept development activities, problem-based case studies, CURE lab experiences, etc.). Again, if the authors choose to not to acknowledge these other classroom practices that promote 3DL, it will in my opinion significantly weaken the paper.

***Response:*** *We agree with the reviewer and have added statements to this effect in the narrative, emphasizing that the use of “active learning” as a catchall for all methods of student engagement is problematic.*

**Reviewer #4:** The authors addressed the review comments thoroughly in the response. No further major and minor revisions are suggested for this article to be considered for full publication.

**Reviewer #7:** I agree with the authors that there may be a confusion about the definition of active learning, but I do not feel that the discussion may be dismissed just by saying that, especially if you are using a definition of AL to validate your work (in terms of the criteria to code segments). The authors consider AL to just be a number of in-class tasks, but Prince (2004) describes it as “any instructional method that engages students in the learning process. In short, active learning requires students to do meaningful learning activities and think about what they are doing”. How do the authors incorporate the reflexive nature of AL in your model, for instance in the example presented in table 1?

***Response:*** *We used the definition of active learning found in the most cited study (Freeman et al. – PNAS, 2014, 111(23, 8410-8415) on the impact of active learning (that is activities that engage students – discussion, clickers, group activities and so on – just as Prince describes). We use these criteria (which are very similar to the COPUS criteria,* <https://doi.org/10.1187/cbe.13-08-0154>*) to characterize what students and faculty are doing in the classroom. Our point is that it matters what students are learning and what they are doing with their knowledge. We hope that this has been made clearer now. We have also emphasized that 3DL is not the only approach to engaging students in authentic scientific practice, and that many transformation efforts may align with 3DL. That is 3DL is an overarching framework that can connect disparate transformation approaches that involve students using knowledge to predict, explain, model and so on.*

Finally, I have still two minor questions. There was no answer to my question on if the inter rater reliability of coding was assessed. I think this information should be in the methodology.

***Response:*** *As we note in the manuscript “Each video was coded by at least two disciplinary experts, and if the IRR fell below 70% a third disciplinary expert also coded the videos. Eventually all videos were coded until all the coders agreed.” The full description of the development and validation of the 3D-LAP were discussed in a prior published report, as noted in the manuscript.*

And again, I do not interpreter the title as the authors seem to do and it seems that to me that it goes beyond what is discussed in the article, suggesting that Al is not as scientifically authentic.

***Response:*** *Again, as many authors have noted, the term active learning can mean many things – one of our goals is to encourage researchers and practitioners alike to better specify what is meant, which is also one of the findings in the large study on Active Learning (*[*https://journals.sagepub.com/doi/10.1177/1529100620973974*](https://journals.sagepub.com/doi/10.1177/1529100620973974)*). Currently the term is a catchall, that can mean many different things (for example at a recent conference it was pointed out that some faculty thought that showing a video clip in class constituted active learning), and as we have found, activities that fall within this term may not engage students in the use of knowledge in an authentic way*

**Reviewer #8:** I wish the connection between the "love" of a subject and how it is taught could be addressed via 3DLearning. I think that professors or teachers who want the best for their students would be flexible in how material is presented and thus assimilated by students.

***Response:*** *As we noted in the prior set of responses, this is an important topic, but we have no data from our studies that would allow us to make this claim. It is beyond the scope of the report.*

7. PLOS authors have the option to publish the peer review history of their article ([what does this mean?](https://urldefense.com/v3/__https:/journals.plos.org/plosone/s/editorial-and-peer-review-process*loc-peer-review-history__;Iw!!HXCxUKc!26WsOKcRWq02djACVd-R2c5q8eWzJ9-MbDu6phbWTeC9jtkZS9s5LLBkJNIETK-LowgqC-Q945OC$)). If published, this will include your full peer review and any attached files.


If you choose “no”, your identity will remain anonymous but your review may still be made public.


**Do you want your identity to be public for this peer review?** For information about this choice, including consent withdrawal, please see our [Privacy Policy](https://urldefense.com/v3/__https:/www.plos.org/privacy-policy__;!!HXCxUKc!26WsOKcRWq02djACVd-R2c5q8eWzJ9-MbDu6phbWTeC9jtkZS9s5LLBkJNIETK-LowgqC69KXjt1$).

Reviewer #1: No

Reviewer #2: No

Reviewer #3: No

Reviewer #4: No

Reviewer #7: No

Reviewer #8: No

[NOTE: If reviewer comments were submitted as an attachment file, they will be attached to this email and accessible via the submission site. Please log into your account, locate the manuscript record, and check for the action link "View Attachments". If this link does not appear, there are no attachment files.]

While revising your submission, please upload your figure files to the Preflight Analysis and Conversion Engine (PACE) digital diagnostic tool, [https://pacev2.apexcovantage.com/](https://urldefense.com/v3/__https:/pacev2.apexcovantage.com/__;!!HXCxUKc!26WsOKcRWq02djACVd-R2c5q8eWzJ9-MbDu6phbWTeC9jtkZS9s5LLBkJNIETK-LowgqC4v5ajIM$). PACE helps ensure that figures meet PLOSrequirements. To use PACE, you must first register as a user. Registration is free. Then, login and navigate to the UPLOAD tab, where you will find detailed instructions on how to use the tool. If you encounter any issues or have any questions when using PACE, please email PLOS at [figures@plos.org](mailto:figures@plos.org). Please note that Supporting Information files do not need this step.

*In compliance with data protection regulations, you may request that we remove your personal registration details at any time.* [*(Remove my information/details)*](https://urldefense.com/v3/__https:/www.editorialmanager.com/pone/login.asp?a=r__;!!HXCxUKc!26WsOKcRWq02djACVd-R2c5q8eWzJ9-MbDu6phbWTeC9jtkZS9s5LLBkJNIETK-LowgqCyzUzM5O$)*. Please contact the publication office if you have any questions.*
